# Identification of Epidemiological Traits by Analysis of SARS−CoV−2 Sequences

**DOI:** 10.3390/v13050764

**Published:** 2021-04-27

**Authors:** Bohu Pan, Zuowei Ji, Sugunadevi Sakkiah, Wenjing Guo, Jie Liu, Tucker A. Patterson, Huixiao Hong

**Affiliations:** National Center for Toxicological Research, U.S. Food and Drug Administration, 3900 NCTR Road, Jefferson, AR 72079, USA; Bohu.Pan@fda.hhs.gov (B.P.); Zuowei.Ji@fda.hhs.gov (Z.J.); Suguna.Sakkiah@fda.hhs.gov (S.S.); Wenjing.Guo@fda.hhs.gov (W.G.); Jie.Liu1@fda.hhs.gov (J.L.); Tucker.Patterson@fda.hhs.gov (T.A.P.)

**Keywords:** SARS−CoV−2, COVID-19, genome, sequence, epidemiological trait, phylogenetic analysis, pattern

## Abstract

Severe acute respiratory syndrome coronavirus 2 (SARS−CoV−2) has caused the ongoing global COVID-19 pandemic that began in late December 2019. The rapid spread of SARS−CoV−2 is primarily due to person-to-person transmission. To understand the epidemiological traits of SARS−CoV−2 transmission, we conducted phylogenetic analysis on genome sequences from >54K SARS−CoV−2 cases obtained from two public databases. Hierarchical clustering analysis on geographic patterns in the resulting phylogenetic trees revealed a co-expansion tendency of the virus among neighboring countries with diverse sources and transmission routes for SARS−CoV−2. Pairwise sequence similarity analysis demonstrated that SARS−CoV−2 is transmitted locally and evolves during transmission. However, no significant differences were seen among SARS−CoV−2 genomes grouped by host age or sex. Here, our identified epidemiological traits provide information to better prevent transmission of SARS−CoV−2 and to facilitate the development of effective vaccines and therapeutics against the virus.

## 1. Introduction

In late December 2019, a new coronavirus named severe acute respiratory syndrome coronavirus 2 (SARS−CoV−2), which causes coronavirus disease 2019 (COVID-19), was first reported in Wuhan, China [1,2,3,4]. Since then, the virus has rapidly spread worldwide, resulting in the World Health Organization (WHO) declaring the COVID-19 outbreak as a public health emergency on 30 January 2020 [5] and a global pandemic on 11 March 2020 [6]. There were over 65 million cases of COVID-19 infection and 1.5 million deaths due to the virus globally as of 4 December 2020 [7]. Unprecedented efforts have been made to combat the virus, resulting in increased understanding of disease epidemiology [8], patient symptoms [9], and pharmacological treatments [10]. The large number of SARS−CoV−2 genome sequences currently available enables genomic epidemiology studies to better understand how this virus is transmitted [11].

Data sharing has long been practiced in the scientific community and it is a fundamental requirement for public health action [12]. A large amount of SARS−CoV−2 sequence data has been generated and shared in several public databases to accelerate research on COVID-19. The GenBank database of the National Center for Biotechnology Information (NCBI) at the National Institutes of Health (NIH) (https://www.ncbi.nlm.nih.gov/genbank/sars-cov-2-seqs/, accessed on 15 September 2020) and the GISAID (Global Initiative on Sharing Avian Influenza Data) database (https://www.gisaid.org/, accessed on 15 September 2020) are currently the two major public databases for SARS−CoV−2 genome sequences. By 24 August 2020, GenBank and GISAID contained 16,683 and 83,699 SARS−CoV−2 sequences, respectively, from contributions made by global clinicians and researchers since December 2019. In addition to virus sequences, both databases also collect basic meta-information, such as sample collection date, host age, and geographic region. These databases provide the scientific community with data that can be used to understand the epidemiological traits of SARS−CoV−2 so that prevention of SARS−CoV−2 transmission can be improved.

The initial full-genome sequence analysis of SARS−CoV−2 classified it as a β-CoV of group 2B with a 30-kb genome [13]. Further phylogenetic analysis revealed its 80% identity to SARS-CoV-1 and 50% identity to MERS-CoV [14,15], both of which were causative agents in previous epidemics [16,17] and possibly originated in bats [18,19]. The phylogenetic analysis of 160 SARS−CoV−2 genomes demonstrated that phylogenetic trees could be used for tracing virus infection sources, which can then be used for improved prevention of COVID-19 viral spread [20]. Phylogenetic analysis of SARS−CoV−2 genomic sequences was also used to identify the introduction and transmission of SARS−CoV−2 in New York City and Brazil [21,22]. These studies revealed that phylogenetic analysis of virus genomic sequences can provide the capacity to understand the evolution and impact of COVID-19.

The publicly available SARS−CoV−2 genomic sequences and associated meta data provided a great opportunity for identifying potential relationships between molecular characteristics and meta factors to better understand the pandemic in a broad view. Rambaut et al. proposed a dynamic nomenclature for naming lineages in the phylogenetic tree constructed from SARS−CoV−2 genome sequences [23]. Mercatelli and Giorgi detected and annotated all mutations in 48,635 SARS−CoV−2 sequences in the GISAID database by comparing with the reference Wuhan genome and found that the major mutational type is single nucleotide transitions, and some clads of sequences have geographic and genomic specificity [24]. Arevalo et al. identified five major haplotypes of 171,461 SARS−CoV−2 genomes in the GISAID database through normalization by relative mutation frequencies and found that the haplotypes are associated with the temporal and geographic distribution, but not with age, gender, or patient status [25]. Shen et al. also found that the haplotypes are associated with a localized transmission pattern at the city, state and country level [26]. These studies demonstrated mutations have distinct geographical patterns and confirmed the relationship between haplotypes and local spread of SARS−CoV−2. However, questions remain regarding other epidemiological traits such as the whole sequence change in genome and in geography, the global transmission pattern or the influence of host genomes and environments on the evolution of the virus genome.

We query about the coevolution between expansion and virus genome sequence under the vision of epidemical features such as geography, collection time and host factors. Are the virus genomes from different geographic locations similar or distinct due to different mutations? What is the evolution trend of virus sequence over time? Do host factors affect virus genomes? To identify these epidemiological traits of COVID-19, we analyzed the large number of genomic sequences of SARS−CoV−2 contained in GenBank and GISAID databases. The phylogenetic trees that we constructed using 12,918 and 42,043 sequences of SARS−CoV−2 in GenBank and GISAID, respectively, revealed several important epidemiological traits of COVID-19. Our analysis provides clear evidence of multiple-origin and domestic expansion for SARS−CoV−2 introductions in many individual countries. Co-expansion in neighboring countries was also observed for the three continents with large numbers of sequences (Europe, Asia, and North America). All of these observations raise the call for a unified, world-wide collaborative effort to combat this global pandemic.

## 2. Materials and Methods

### 2.1. Data Collection

On 15 November 2020, SARS−CoV−2 sequences (23,735 from GenBank and 94,201 from GISAID) were downloaded. The sequences were in DNA format converted from the plus RNA sequences of SARS−CoV−2. Corresponding metadata with sequence ID as a unique key for these sequences were also downloaded from the databases at the same time. Metadata from the two databases are summarized in Supplementary Table S1, and the detailed metadata along with the sequences used in this study are available upon request.

### 2.2. Data Preprocessing

Prior to phylogenetic analysis, the downloaded data were preprocessed. Sequence quality was assessed by counting the number of “N” in each sequence. We examined the distribution of sequence lengths for both datasets (Supplementary Figures S1 and S2). To avoid uncertainty in sequence similarity caused by “N”, sequences that contained a high number of “N” or that were shorter than 29,500 bases were excluded, resulting in 12,918 and 42,043 sequences from GenBank and GISAID, respectively, for subsequent phylogenetic analysis. Data preprocessing was conducted using in-house python scripts.

### 2.3. Phylogenetic Analysis

Phylogenetic analysis was performed with multiple sequence alignment, format transfer, and phylogenetic tree construction and display. First, sequences were aligned to the reference genome of SARS−CoV−2 from GenBank (NC_045512.2) using the multiple sequence alignment program MAFFT [27] (https://mafft.cbrc.jp/alignment/software/mafft-7.467-without-extensions-src.tgz, accessed on 15 September 2020) (Kazutaka Katoh, Osaka University, Osaka Prefecture, Japan). The running parameter was set as “-auto -thread −1 -keeplength -addfragments” with aligned fasta as default output. The multiple sequence alignment results were converted to PHYLIP format using the alignment transformation tool ALTER [28] (version: 1.3.4-jar-with-dependencies). Phylogenetic analysis was performed on the sequence alignment results in PHYLIP format using FastTree [29] (version: 2.1.11) with approximately-maximum-likelihood and generalized time-reversible (GTR) models for nucleotide evolution. The online tool iTOL (Interactive Tree of Life) [30] was used to display the phylogenetic trees with circus model and the clades were annotated in colors.

### 2.4. Clustering Analysis

Major clades consisting of similar sequences in the generated phylogenetic trees were formed according to distance of sequences to the root and with sizes of 50–1500 and 200–3500 sequences for the GenBank and GISAID datasets, respectively, using an in-house Python script and the Python library ETE TOOLKIT [31] (v3.0). Distributions of metadata for sequences in the major clades were used for clustering analysis to examine the relationship between virus genomes and metadata. Metadata were explored by clustering analysis which included geographical location (country), time (date), patient age, and sex for each virus sample. Original country names were replaced with their three-letter codes following the ISO 3166 international standard and the corresponding replacement was recorded in Supplementary Table S2. Collection dates were grouped by months, with the samples collected before 2020 combined with samples collected in January 2020 in the “January and Before” group due to the low number of samples collected for sequencing during the early stages of the pandemic. Patient age and sex were explored only for the GISAID dataset because GenBank did not include patient age and sex for its sequences. Patient age was divided into four groups: youth (<18 years), young adult (18–35 years), adult (36–55 years), and senior (>55 years). Hierarchical clustering was performed on distributions of virus genomes in the clades for the groups of metadata using the “Clustermap” function in python package “seaborn” (v.0.10.1, https://seaborn.pydata.org/generated/seaborn.clustermap.html accessed on 23 April 2021) (Michael Waskom, New York University, New York, NY, USA).

### 2.5. Sequence Similarity Analysis

In addition to the clustering analysis on the distribution of the virus sequences in the major clades of the constructed phylogenetic trees from multiple sequence alignments, pairwise sequence similarities between the virus genomes were calculated to investigate epidemiological traits of SARS−CoV−2. Similarity between two virus genomes was measured using the distance between the two sequences; pairwise distances for all sequences in GenBank and GISAID were calculated separately.

We calculated pairwise sequence distances between all sequences using Mothur [32]. Multiple alignment files from MAFFT were used as the input file for dist.seqs module in Mothur. A string of gaps was taken as one gap for the distance calculation by default setting. Both mismatch and gap were penalized for distance calculation. Multiple processors were used in the calculation and the lower triangle portion of the distance matrix was set for output format. The output distances were incorporated and classified with their meta information using in-house python scripts for comparative analysis.

## 3. Results

### 3.1. Phylogenetic Tree

**Figure 1:**
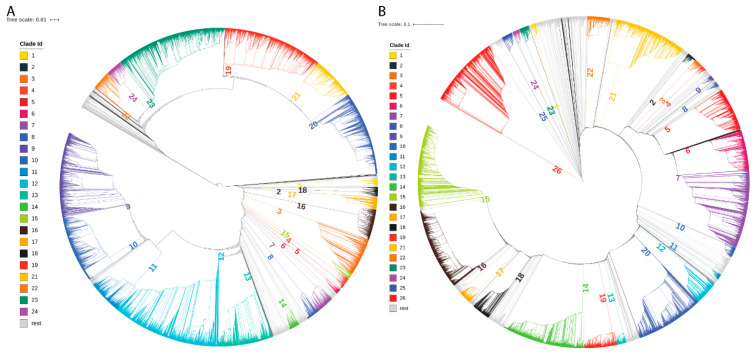
Phylogenetic analysis results. (**A**) Phylogenetic tree from the GenBank dataset. (**B**) Phylogenetic tree from the GISAID dataset. The major clades are marked in colors and labeled with numbers.

After preprocessing, 12,918 and 42,043 virus genome sequences from GenBank and GISAID were used in phylogenetic analysis, respectively. In total, there were 160 and 379 clades formed in the phylogenetic trees constructed for GenBank and GISAID sequences, respectively. The clades with very few sequenced were removed and the remaining clades were defined as major clades and used in subsequent characteristics analysis. The 24 and 26 major clades (Supplementary Tables S3 and S4) were formed by 11,887 (92%) sequences from GenBank and 34,632 (82.4%) sequences from GISAID, respectively, and were marked by different colors in the obtained phylogenetic trees (Figure 1). For most of the clades, most of the sequences were from the same country. For example, all sequences in clades 1, 4, 5, and 6 were from the USA and all sequences in clades 19, 20, and 21 were from Australia in the phylogenetic tree from GenBank data (Figure 1A); 96.3%, 96.5%, 86.6%, and 72.9% sequences in clades 25, 2, 16, and 9 are from the USA, United Kingdom, Australia, and the Netherlands, respectively, in the phylogenetic tree from GISAID data (Figure 1B). Some large clades contained sequences from many countries. However, most of the sequences in such clades were from the same country. For example, clade 23 in the phylogenetic tree from GenBank data contained 1498 sequences from 36 countries; but most of them (70.6%) were from the USA (Figure 1A), and clade 5 in the phylogenetic tree from GISAID contained 1796 sequences from 42 countries; but most of them (72.4%) were also from the USA (Figure 1B). The major clades in the phylogenetic tree from GISAID sequences are in a good correlation with the lineages recorded in GISAID (Supplementary Table S5).

Examination of the geographic locations for sequences in the major clades revealed that virus sequences from the same countries were grouped together in the same clades (Figure 1, Supplementary Tables S3 and S4). For example, in the phylogenetic tree generated from GenBank data (Figure 1A), 94.1% of the sequences from Egypt were grouped in clade 10; 85.7% of the sequences from Bangladesh were grouped in clade 22; 93.6% of the sequences from India were grouped in clades 9 and 10; 70.9% of the sequences from Australia were grouped in clades 19 and 20; and 36.4% sequences from the USA were grouped in clades 12 and 23. In the phylogenetic tree generated from GISAID data (Figure 1B), 89.4% of the sequences from Australia were grouped in clade 6 and 64.0% of the sequences from South Korea were grouped in clade 22.

### 3.2. Diverse Sources of Transmission

**Figure 2:**
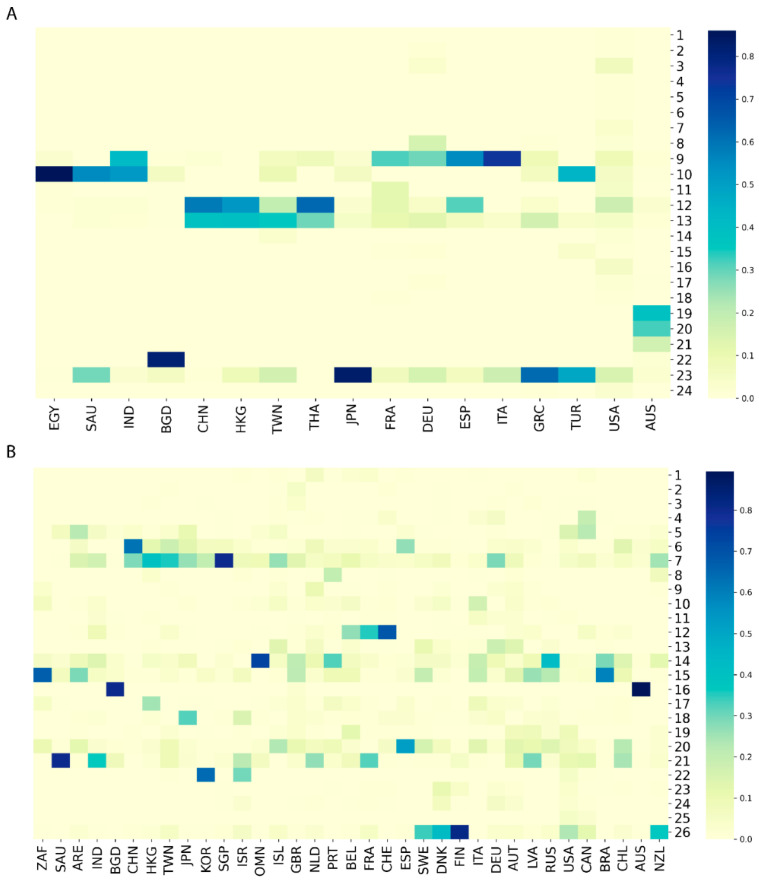
Distributions of SARS−CoV−2 sequences in the major clades for countries from GenBank (**A**) and GISAID (**B**). Each column represents a country which is labeled with a three-letter country code. Each row depicts a major clade marked by its clade number. The color palette indicates frequency values of sequences in the major clades for individual countries.

The phylogenetic trees revealed that virus genomes within the same countries were more similar than those between countries, but the distribution patterns of the virus sequences were different among countries. To more clearly estimate the sources and routes of SARS−CoV−2 transmission, we examined distributions of virus sequences in the major clades among countries by hierarchy clustering analysis. Hierarchy clustering results for both GenBank data (Supplementary Figure S3A) and GISAID data (Supplementary Figure S3B) showed that neighboring countries had similar distribution patterns in the major clades (e.g., 9 and 12 in Supplementary Figure S3A and 12 and 15 in Supplementary Figure S3B) of the phylogenetic trees, indicating that genomic diversity was similar for the neighboring countries, and that transmission occurred more readily among neighboring countries than among countries that are geographically disconnected. To examine the geographical pattern of sequence distribution in the major clades, countries from the same continent were grouped together and the neighboring countries were connected in the same order for GenBank and GISAID (Figure 2). For example, most of the SARS−CoV−2 sequences from China, Hong Kong, and Taiwan were in clades 12 and 13 of GenBank (Figure 2A) and clades 6 and 7 of GISAID (Figure 2B). Similarly, most virus sequences in the GISAID from the neighboring countries Belgium, France, and Switzerland were in clade 12 and from Sweden, Denmark, and Finland in Clade 26 (Figure 2B). This conclusion can also be supported by the phenomenon observed in GenBank. Most sequences from the neighboring European countries France, Germany, Spain, and Italy were in clades 9 (Figure 2A). Moreover, we found that each individual country may show diverse sources of SARS−CoV−2. According to our analysis, by calculating the frequency of samples in each clade over the total samples from that country, for countries with more than twenty samples in GenBank, the majority of frequencies in their corresponding clades were less than 25% (Supplementary Figure S3A and Supplementary Table S3). In parallel, the majority of frequencies were also less than 25% for the samples in GISAID (Supplementary Figure S3B and Supplementary Table S4). Because genomic sequences from most countries were discretely distributed in multiple clades in both datasets, it is reasonable to infer that most countries have had multiple origins for SARS−CoV−2. This deduction can also be proven by comparing frequency distributions between countries. Taking France as an example, it showed a similar frequency distribution pattern with Germany in the GenBank dataset (Figure 2A), which indicates that these countries may have had similar virus sources. However, in the GISAID dataset (Figure 2B), France was observed to be more similar to Belgium than to Germany. This inconsistency in the two datasets could be explained by the uneven distribution or diverse sources of SARS−CoV−2 within one country.

### 3.3. Dynamic Evolution of SARS−CoV−2 across Time

**Figure 3:**
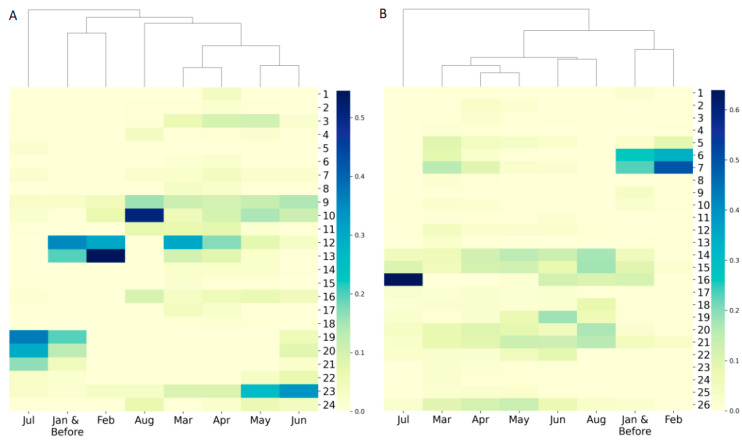
Hierarchical clustering of sequence frequency distribution in sequencing collection time period using (**A**) the GenBank dataset, and (**B**) the GISAID dataset. Each column represents a sequence collection time period shown as an x-axis tick label. Each row depicts a major clade marked by its clade number. The color palette indicates the sample frequency.

To gain insight into the pattern of virus variation over time, the distribution of virus genomes in the major clades based on the time of sample collection was also investigated by hierarchy clustering. As shown in Figure 3, clustering for collection time could be divided into three groups: the first group consists of sequences collected in July, the second group contains the sequences collected in February and before, and the last group has sequences collected in March to June as well as in August. The cluster of February and before indicates consistency of expansion during this period. For the group of March to June plus August, the clustering reflects that the coronavirus genome has been changing over time, which is consistent with its mutation nature. In the case of the first group of July, the expansion was primarily distributed in clades 19, 20, and 21 for the GenBank dataset and clade 16 for the GISAID dataset (Figure 3, Supplementary Tables S6 and S7). The sharp change in consecutive months in specific clusters observed here which reveal SARS−CoV−2 genome evolution in specific countries should remind researchers to exercise great caution with vaccine design.

**Figure 4:**
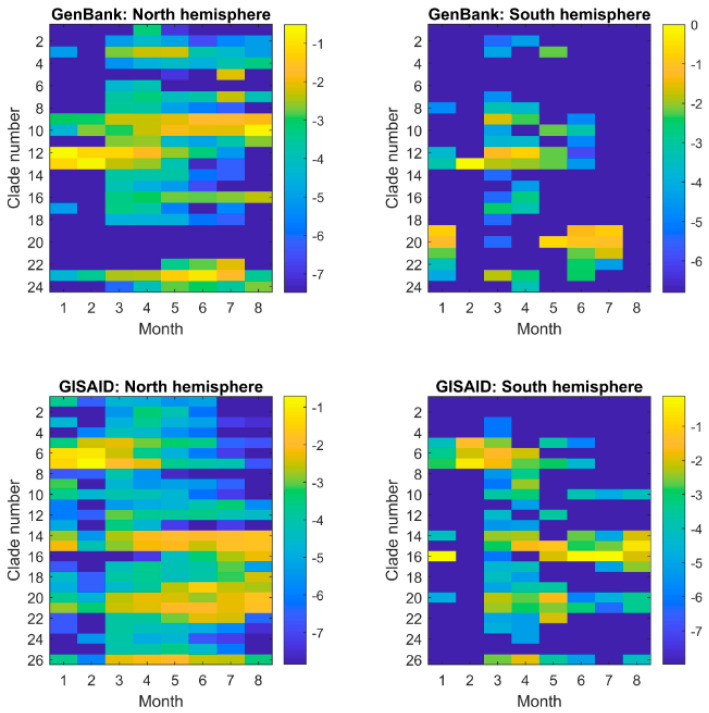
Similarity pattern of SARS−CoV−2 sequences collected in different periods of time (x-axis) in GenBank (the **top** panel) and GISAID (the **bottom** panel) from the countries in the north hemisphere (the **left** panel) and south hemisphere (the **right** panel). The fractions of the sequences collected in a month in the major clades were represented by the logarithmic values of the numbers of sequences in major clades divided by total number of sequences collected in the month and depicted by the color legend at the right side. No sequence was in the major clades from the countries in the south hemisphere for August in GenBank, and thus the minimum was used to represent the corresponding logarithmic values.

We examined the similarity patterns (distributions in the major clades) of sequences collected in different time periods (Figure 4). Interestingly, comparing the sequencing similarity patterns of collection months did not reveal significant difference between GenBank and GISAID databases (the top panel versus the bottom panel in Figure 4), while the sequences collected from the countries in the north hemisphere had substantial differences from the sequences collected from the countries from the south hemisphere (the left panel versus the right panel) for both GenBank and GISAID. For the same month temperature is quite different between the south hemisphere and north hemisphere, the difference in sequences might be associated with the temperature differences. Our results indicate that the SARS−CoV−2 virus might evolve in different ways to interact with environmental factors such as temperature.

### 3.4. Host Age and Sex

Because obvious variations in genome sequences were observed in the above analysis, we questioned whether different host attributes (e.g., age and sex) are associated with the variation in virus genomes. Due to the lack of patient age and sex information in the GenBank dataset, only the GISAID dataset was used to examine possible effects from host age and sex (Supplementary Figure S4, Supplementary Table S8). Although, as expected, the youth group (<18 years) stood out, no significant variation of the frequency in different clades was observed among the other age groups examined. Likewise, we did not detect noticeable impacts from host sex because the frequency distribution pattern in most clades was similar between males and females (Supplementary Figure S5, Supplementary Table S9).

### 3.5. Pairwise Sequence Similarity Analysis

**Figure 5:**
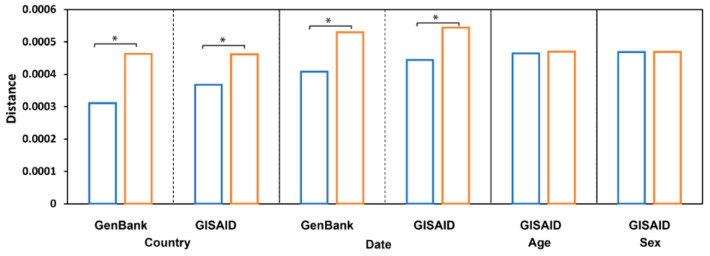
Pairwise sequence distances. The average distance between sequences from the same groups (blue bars, from sequence comparison within the same country/date/age/sex) and from different groups (orange bars, from sequence comparison of different country/date/age/sex) is depicted at the y-axis. Sequences are grouped by countries, sample collection time, patient age, and patient sex (the x-axis label). The groups with significant differences, *p* < 0.05, were labeled with stars (*).

Distances between the viral genomic sequences within countries were significantly shorter than those between the viral genomic sequences across countries, with *p* values less than 0.0001 for both the GenBank and GISAID datasets (Figure 5). The pairwise sequence similarity analysis suggested that SARS−CoV−2 is primarily transmitted locally, because its sequence exhibited significant variability among countries in both databases. This, in turn, confirms the role of geography in the sequence variation of virus genome in our hierarchical analysis.

To examine variation in the virus sequences collected on different dates, we compared distances between virus sequences collected in the same month with sequences collected in different months and found that they were significantly different, *p* value = 0.0056 and 0.0238 (paired two-tail Student′s *t*-Test) for the sequences in GenBank and GISAID, respectively (Figure 5), indicating a rapid evolution of the SARS−CoV−2 genomic sequence.

In contrast to geographic location and collection date, no observable differences were found when comparisons were made among SARS−CoV−2 genomes grouped by host age or sex (Figure 5). Distances between sequences collected from patients in the same age group (mean = 4.65 × 10^−4^, standard deviation (std) = 1.81 × 10^−5^) were not significantly different (*p* = 0.419) from distances between sequences collected from patients in different age groups (mean = 4.70 × 10^−4^, std = 7.13 × 10^−6^); distances between sequences collected from patients of the same sex (mean = 4.69 × 10^−4^, std = 4.24 × 10^−6^) were not significantly different (*p* = 0.910) from distances between sequences collected from patients of different sexes (mean = 4.70 × 10^−4^, std = 7.07 × 10^−7^), indicating that host age and sex do not affect mutations in the SARS−CoV−2 genome. Thus, the observed morbidity and mortality differences among people at different ages and of different sexes should not be explained by virus genome differences but may be due to human genetic and metabolic diversity. Other factors regarding host attributes warrant further investigation.

## 4. Discussion

Data and information sharing enabled by public databases have made it possible for scientists to better understand and manage public health emergencies such as the ongoing COVID-19 pandemic [12,33]. However, poor data quality hinders the progress of scientific research. For instance, only 13 of 60 countries were represented by more than 30 high-quality sequences in the GenBank database. Although the number of sequences in the GISAID database was large, only ~40% of the sequences met our data quality filter criteria. In addition, limited meta information is contained in public databases, which is likely due to the difficulty posed by summary and collection [34]. Moreover, it should be noted that some underdeveloped countries may have limited technologies for testing and sequencing; the majority of sequences in both datasets primarily came from developed countries [35].

Phylogenetic analysis is a popular method used in genetic studies and is computationally demanding for long-sequence alignments. “Maximum likelihood” is the most popular algorithm for inferring a phylogenetic tree and its confidence values [36]. On the large datasets of SARS−CoV−2 genomes in our study, we used a specific version of MAFFT [27] for long-sequence alignment, and FastTree [29], which is a faster and more accurate tool for phylogenetic analysis due to its elimination of O(N2) steps in the neighbor-joining phase. More efficient tools are needed for phylogenetic analysis because the number of SARS−CoV−2 sequences available for phylogenetic analysis are dramatically increasing. Another difficult task is to identify the optimal number of clades for a phylogenetic tree constructed from a large amount of genome sequences. We generated clades from the phylogenetic trees here with two major aims: sizes of the clades should not be too large, and the number of major clades should not be too small. This is a limitation of this study because different optimal number of clades and major clades could be generated for the two datasets.

In this study, our hierarchical clustering analysis of geography in the resulting phylogenetic trees demonstrated the tendency of SARS−CoV−2 to co-expand among neighboring countries. Moreover, diverse sources and transmission routes of SARS−CoV−2 were observed for most of the countries analyzed. In the case of the USA, the country displayed domestic and multi-origin expansion in both datasets. The multi-origin expansion pattern in the GISAID dataset was more evident compared to the GenBank dataset, which is a reasonable outcome because data were more abundant in the GISAID dataset. Collectively, based on our data, it appears that restrictions on local and international travel may assist in curtailing the spread of the virus. In addition, documentation of individual travel histories would facilitate contact tracing and the determination of points of origin. Given the identified attribute of rapid mutation for SARS−CoV−2 based on our collection date analysis, global collaboration among scientific communities should be strengthened to facilitate swift and appropriate action. Improvements in intervention and therapeutic development can be made only with timely updated information on the virus and its effects. Taken together, the results of our epidemiological investigation showed that this coronavirus exhibits a high degree of spatial and temporal variability, which indicates that a global effort is required to fight against this global pandemic.

## 5. Disclaimer

The content is solely the responsibility of the authors and does not necessarily represent the official views of U.S. Food and Drug Administration.

## Data Availability

The source sequence data and corresponding meta information are downloaded from two public databases: GenBank and GISAID, which will be available upon request.

## Supplementary Materials

The following are available online at https://www.mdpi.com/article/10.3390/v13050764/s1, Figure S1: Sequence length distribution of GISAID set, Figure S2: Sequence length distribution of GenBank set, Figure S3: Hierarchical clustering of sequence frequency distribution in countries, Figure S4: Hierarchical clustering of sequence frequency distribution in host age groups of GISAID dataset, Figure S5: Hierarchical clustering of sequence frequency distribution in host genders of GISAID dataset, Table S1: Summary of meta information of GenBank and GISAID datasets, Table S2: Country names and their three-letter codes, Table S3: Sequence distribution in the major clades of GenBank dataset by countries/regions, Table S4: Sequence distribution in the major clades of GISAID dataset by countries/regions, Table S5: Association between clades and PANGO lineages, Table S6: Sequence distribution in the major clades of GenBank dataset by collection date groups, Table S7: Sequence distribution in the major clades of GISAID dataset by collection date groups, Table S8: Sequence distribution in the major clades of GISAID dataset by host age groups, Table S9: Sequence distribution in the major clades of GISAID dataset by host genders.
