# Supplementary material for: Identification of Epidemiological Traits by Analysis of SARS−CoV−2 Sequences
Source: Viruses. 2021 Apr 27;13(5):764. doi: 10.3390/v13050764 (PMC8145049; doi:10.3390/v13050764)
Supplement: Supplementary file 1 [file viruses-13-00764-s001.zip › viruses-1141707-supplementary.pdf]

Supplementary Material:

Identification of epidemiological traits by analysis of SARS-CoV-2 sequences

Bohu Pan[†], Zuowei Ji[†], Suguna Sakkiah, Wenjing Guo, Jie Liu, Tucker A. Patterson, Huixiao Hong*

National Center for Toxicological Research, U.S. Food and Drug Administration, 3900 NCTR Road, Jefferson, AR 72079, USA

† These authors made equal contributions

*Correspondence should be addressed to Dr. Huixiao Hong: huixiao.hong@fda.hhs.gov

Table of content:

Supplementary Figure S1. Sequence length distribution of GISAID set. **p3**

Supplementary Figure S2. Sequence length distribution of GeneBank set. **p4**

Supplementary Figure S3. Hierarchical clustering of sequence frequency distribution in countries. **p5**

Supplementary Figure S4. Hierarchical clustering of sequence frequency distribution in host age groups of GISAID dataset. **p6**

Supplementary Figure S5. Hierarchical clustering of sequence frequency distribution in host genders of GISAID dataset. **p7**

Supplementary Table S1. Summary of meta information of GeneBank and GISAID datasets. **p8**

Supplementary Table S2. Country names and their three-letter codes. **p9-10**

Supplementary Table S3. Sequence distribution in the major clades of GeneBank dataset by countries/regions. **p11**

Supplementary Table S4. Sequence distribution in the major clades of GISAID dataset by countries/regions. **p12**

Supplementary Table S5. Association between clades and PANGO lineages. **p13**

Supplementary Table S6. Sequence distribution in the major clades of GeneBank dataset by collection date groups. **p14**

Supplementary Table S7. Sequence distribution in the major clades of GISAID dataset by collection date groups. **p15**

Supplementary Table S8. Sequence distribution in the major clades of GISAID dataset by host age groups. **p16**

Supplementary Table S9. Sequence distribution in the major clades of GISAID dataset by host genders. **p17**

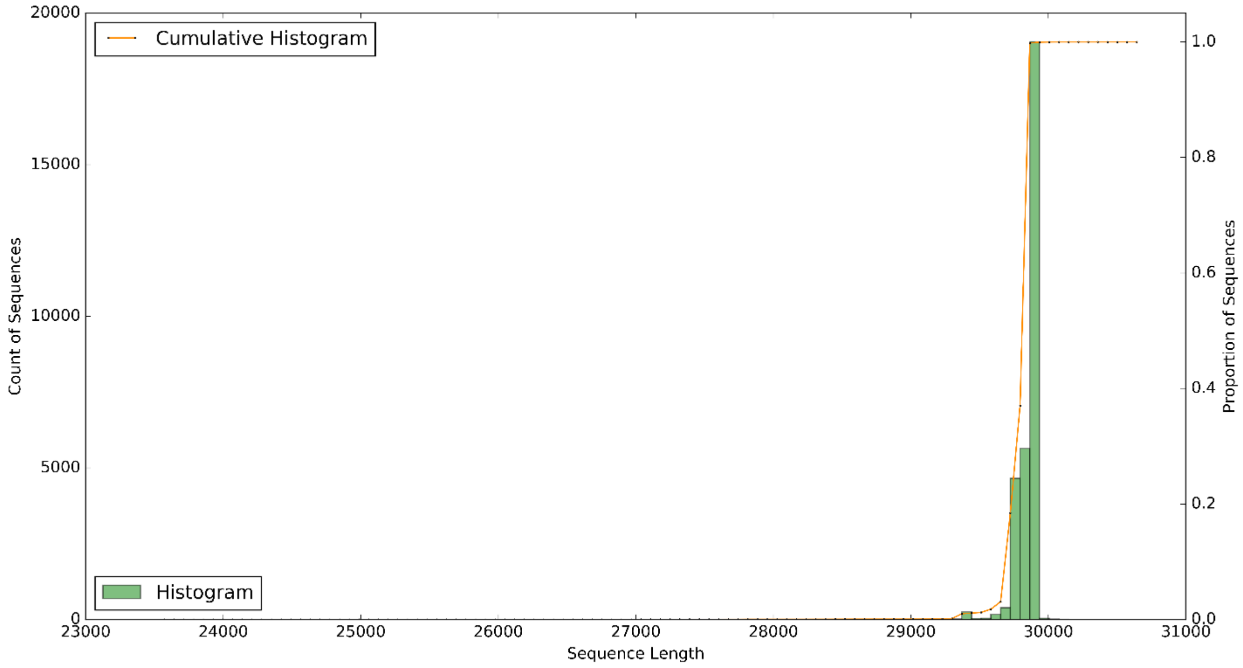

Supplementary Figure S1. Sequence length distribution of GISAID set. The x axis depicts the sequence length of each sequenced genomes. The left y axis depicts the count of corresponding sequences for each sequence. The right y axis depicts the cumulative proportion of the corresponding sequences.

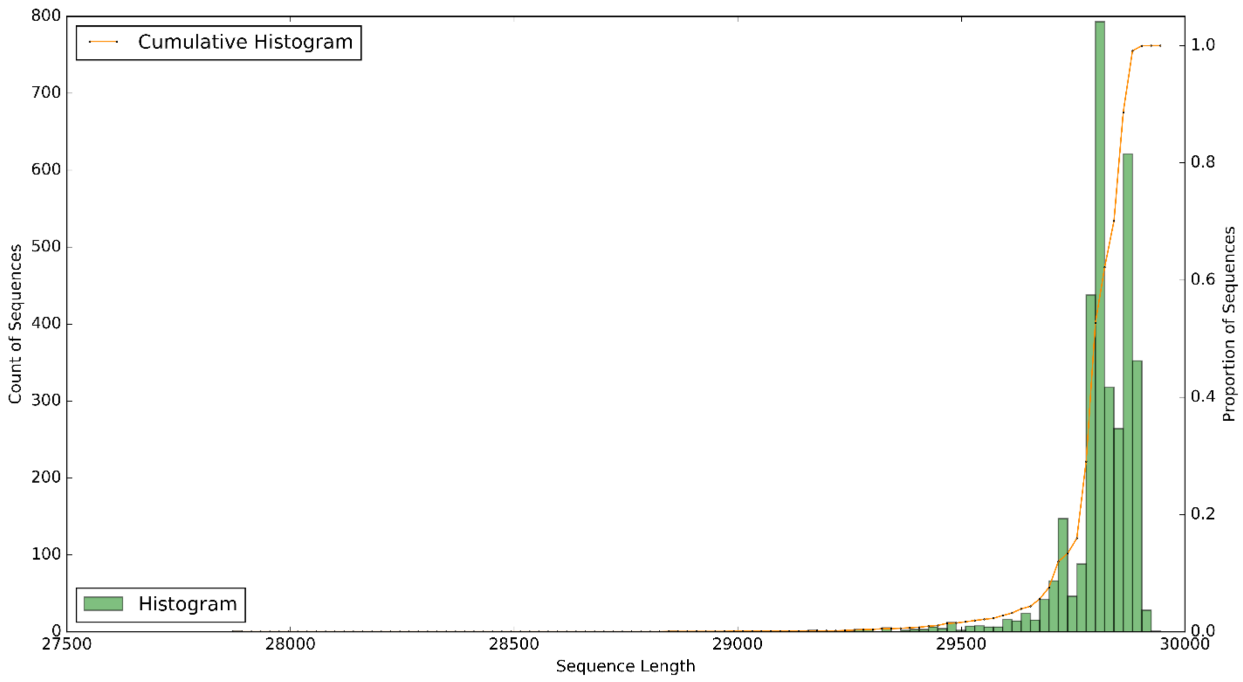

Supplementary Figure S2. Sequence length distribution of GeneBank set. The x axis depicts the sequence length of each sequenced genomes. The left y axis depicts the count of corresponding sequences for each sequence. The right y axis depicts the cumulative proportion of the corresponding sequences.

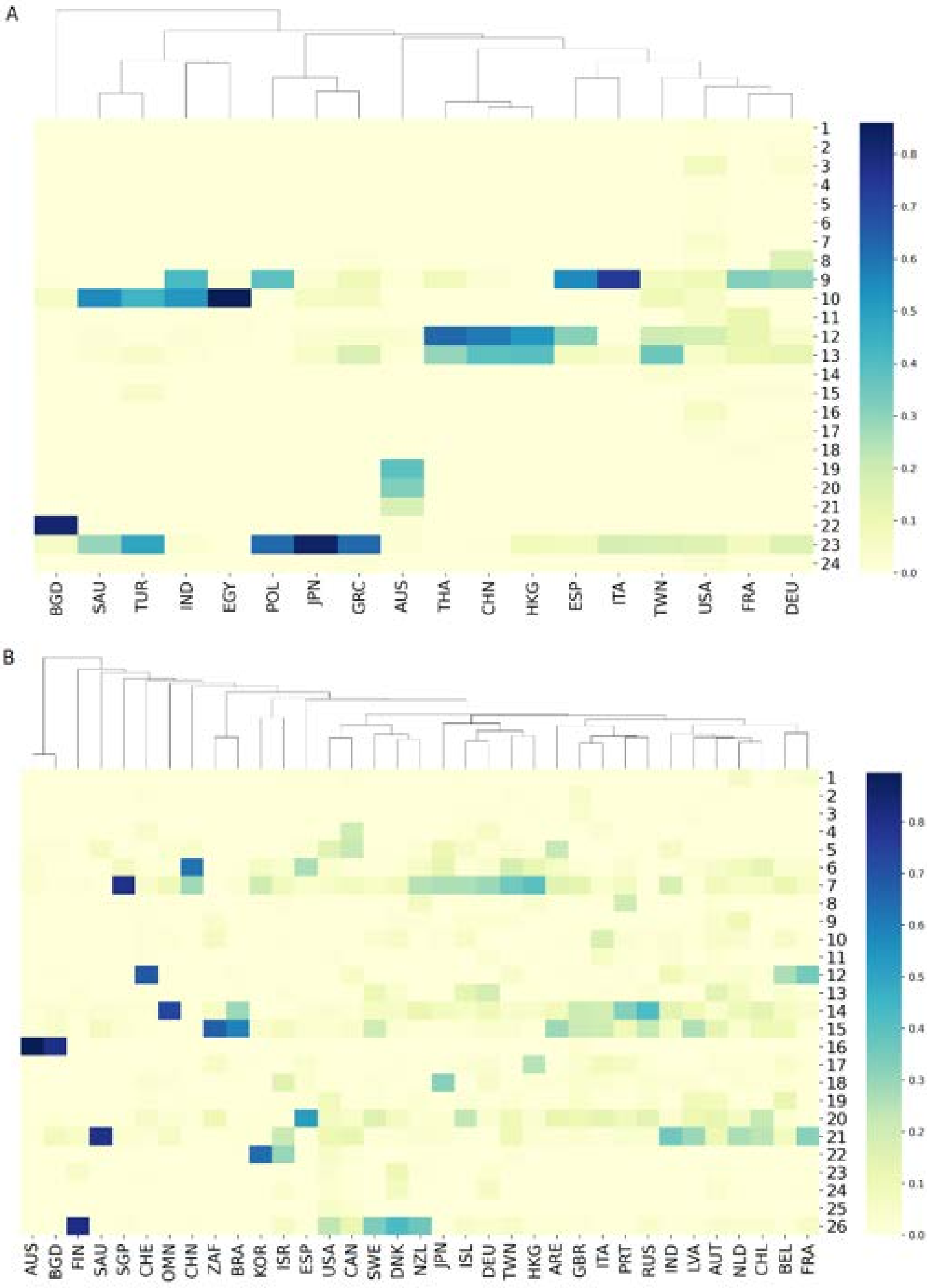

Supplementary Figure S3. Hierarchical clustering of sequence frequency distribution in countries/regions using (**a**) the GeneBank dataset, and (**b**) the GISAID dataset. Each column represents a country which is labeled with a three-letter country code. Each row depicts a major clade marked by its clade number. The color palette indicates the sample frequency.

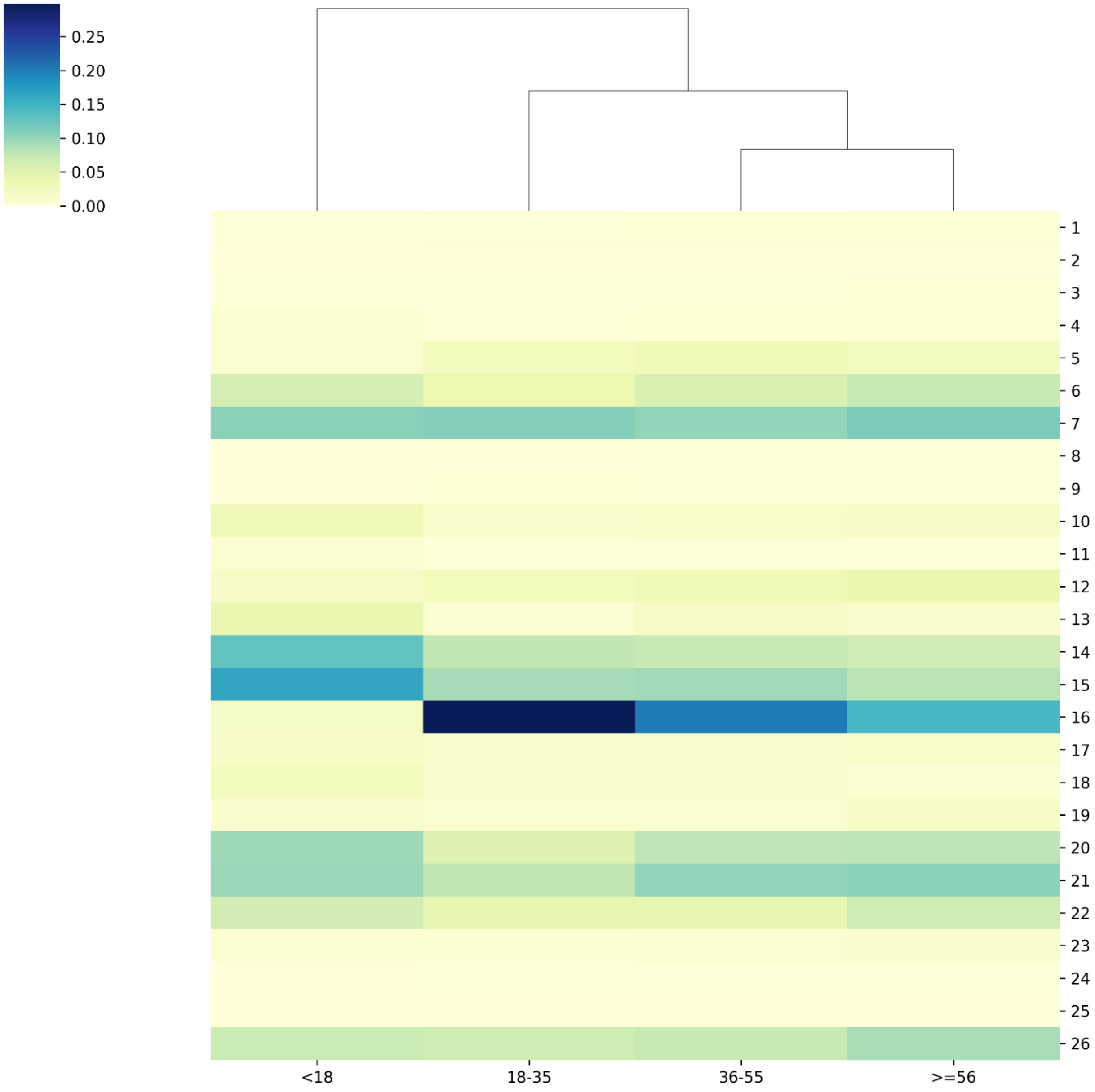

Supplementary Figure S4. Hierarchical clustering of sequence frequency distribution in host age groups of GISAID dataset. Each column represents an age group shown as an x-axis tick label. Each row depicts a major clade marked by its clade number. The color palette indicates the sample frequency.

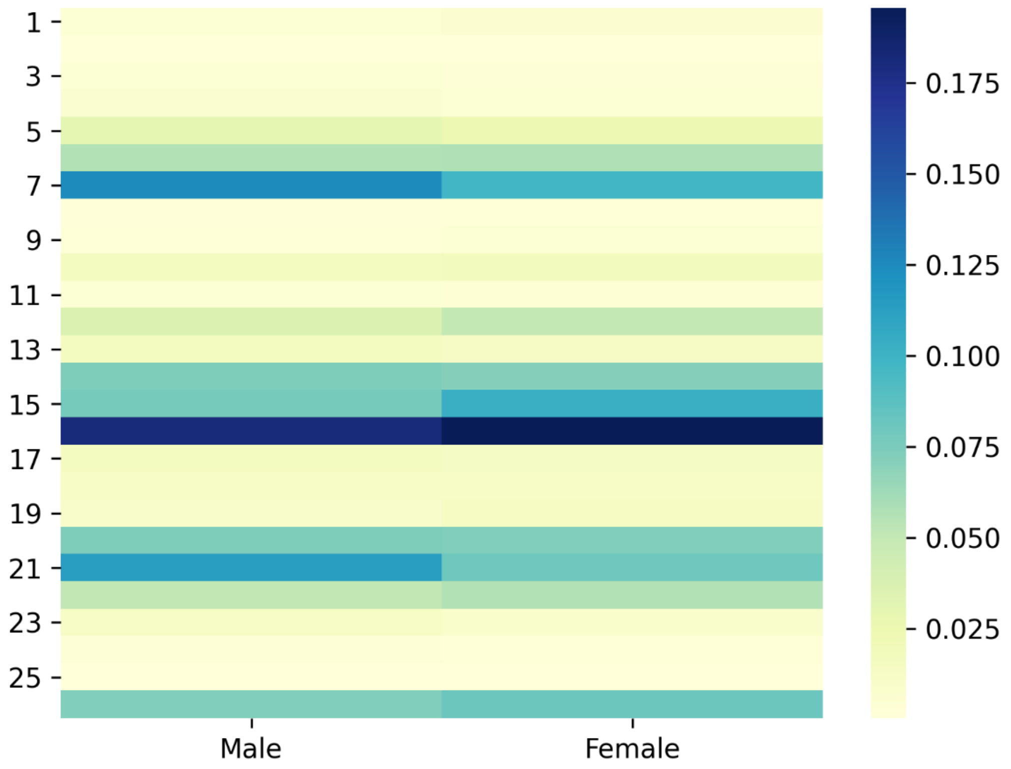

Supplementary Figure S5. Hierarchical clustering of sequence frequency distribution in host Genders of GISAID dataset. Each column represents a gender type shown as an x-axis tick label. Each row depicts a major clade marked by its clade number. The color palette indicates the sample frequency.

Supplementary Table S1. Summary of meta information of GeneBank and GISAID datasets.

**Data Set**		**GeneBank**	**GISAID**
**Sequence Number**		23735	99518
**Selected Sequences**		12918	42043
**Selected Sequences**	**With Country**	12356	40673
	**With Date**	12286	40673
	**With Age**	NA	16047
	**With Gender**	NA	16530

Supplementary Table S2. Country names and their three-letter codes.

**Country Name**	**3 letter code**
Australia	AUS
Austria	AUT
Bangladesh	BGD
Belgium	BEL
Brazil	BRA
Canada	CAN
Chile	CHL
China	CHN
Denmark	DNK
Egypt	EGY
Finland	FIN
France	FRA
Germany	DEU
Greece	GRC
Hong Kong	HKG
Iceland	ISL
India	IND
Israel	ISR
Italy	ITA
Japan	JPN
Latvia	LVA
Netherlands	NLD
New Zealand	NZL
Oman	OMN
Portugal	PRT

Russia	RUS
Saudi Arabia	SAU
Singapore	SGP
South Africa	ZAF
South Korea	KOR
Spain	ESP
Sweden	SWE
Switzerland	CHE
Taiwan	TWN
Thailand	THA
Turkey	TUR
United Arab Emirates	ARE
United Kingdom	GBR
USA	USA

Supplementary Table 3. Sequence distribution in the major clades of GeneBank dataset by countries/regions*

		Africa	Asia								Europe						North America	Oceania
Clade Id	Sequences	EGY	SAU	IND	BGD	CHN	HKG	TWN	JPN	THA	FRA	GRC	DEU	ESP	ITA	TUR	USA	AUS
1	75	0	0	0	0	0	0	0	0	0	0	0	0	0	0	0	75	0
2	57	0	0	0	0	0	0	0	0	0	0	0	1	0	0	0	54	2
3	511	0	0	0	0	0	0	0	0	0	0	0	2	0	0	0	497	4
4	86	0	0	0	0	0	0	0	0	0	0	0	0	0	0	0	86	0
5	61	0	0	0	0	0	0	0	0	0	0	0	0	0	0	0	61	0
6	71	0	0	0	0	0	0	0	0	0	0	0	0	0	0	0	71	0
7	246	0	0	0	0	0	0	0	0	0	0	0	0	0	0	0	240	2
8	175	0	0	0	0	0	0	0	0	0	0	1	10	0	0	0	124	10
9	1144	5	0	201	2	2	2	0	2	0	27	7	19	2	0	17	627	50
10	907	160	0	252	13	0	0	31	5	0	0	5	0	3	10	0	394	19
11	463	2	0	0	1	0	0	0	0	0	10	0	0	0	0	0	352	10
12	1465	2	0	9	1	61	15	1	2	0	10	3	3	6	0	0	1207	102
13	733	0	65	8	1	40	7	1	4	0	9	13	8	11	1	1	352	58
14	140	0	0	0	0	0	0	0	0	0	0	0	0	1	0	0	138	1
15	81	0	0	0	0	0	0	0	0	0	1	0	1	0	1	0	74	1
16	406	0	0	0	0	0	0	0	0	0	0	0	0	0	0	0	400	6
17	170	0	0	1	0	0	0	0	0	0	0	0	1	0	0	0	140	21
18	68	0	0	0	0	0	0	0	0	0	1	0	0	0	0	0	59	1
19	1342	0	0	0	0	0	0	0	0	0	0	0	0	0	0	0	0	1342
20	1134	0	0	0	0	0	0	0	0	0	0	0	0	0	0	0	0	1134
21	582	0	0	0	0	0	0	0	0	0	0	0	0	0	0	0	0	582
22	256	0	0	0	186	0	0	0	0	0	0	0	0	0	0	0	0	70
23	1498	1	0	13	13	1	0	16	70	0	6	49	10	5	11	4	1057	76
24	216	0	0	0	0	0	0	0	0	0	0	1	0	0	0	0	208	3

*See full name in supplementary table 2.

Supplementary Table 4. Sequence distribution in the major clades of GISAID dataset by countries/regions*

		Africa	Asia												Europe																North America		South America		Oceania	
Clade Id	Sequences	ZAF	SAU	ARE	IND	BGD	CHN	HKG	TWN	JPN	KOR	SGP	ISR	OMN	GBR	NLD	PRT	BEL	ESP	SWE	FRA	DNK	RUS	ISL	CHE	ITA	FIN	DEU	AUT	LVA	USA	CAN	BRA	CHL	AUS	NZL
1	211	0	0	0	0	0	0	0	0	0	0	0	1	0	24	93	5	14	7	0	15	0	0	3	0	1	0	0	0	0	15	12	0	0	0	0
2	395	0	0	0	0	0	0	0	1	0	0	0	0	0	381	0	0	0	0	0	0	1	0	2	4	0	0	0	1	0	0	0	0	0	0	0
3	353	0	0	0	1	0	0	1	0	1	3	0	0	0	277	0	2	0	0	0	1	1	0	3	0	0	0	1	0	2	37	3	0	0	0	0
4	261	0	0	0	0	0	0	0	0	0	0	1	0	0	39	15	0	4	0	0	2	1	2	4	10	4	0	5	0	0	25	123	2	0	1	0
5	1796	0	30	31	54	5	26	1	3	60	0	1	0	4	6	5	2	4	13	3	0	0	2	15	0	0	0	1	0	0	1302	138	0	1	37	2
6	1685	1	1	8	3	0	417	12	17	75	44	15	5	2	159	125	23	18	163	7	14	3	1	3	3	0	1	3	6	3	315	7	3	17	84	5
7	3289	0	2	21	160	1	191	40	33	151	136	166	12	16	1032	86	55	71	22	28	22	17	2	76	10	6	3	25	12	2	459	47	7	7	70	23
8	221	0	0	0	1	0	0	4	0	0	0	0	2	0	23	9	156	0	0	0	0	0	0	3	1	1	0	0	2	0	6	0	0	0	1	7
9	218	15	0	0	0	0	0	0	1	0	0	0	0	0	0	159	3	3	0	0	0	4	0	7	3	0	0	2	5	1	5	0	0	0	0	1
10	425	27	0	1	30	0	0	0	0	9	0	0	1	3	59	65	20	36	0	15	4	11	5	16	4	26	1	0	6	1	14	0	0	0	3	0
11	236	1	0	0	23	1	1	1	0	1	0	0	0	0	35	2	7	1	0	0	3	1	2	0	3	8	0	2	3	0	138	0	0	0	0	0
12	1007	1	0	1	85	2	2	0	4	3	1	0	0	1	65	53	13	168	0	0	141	1	1	0	163	1	1	0	3	5	13	33	5	3	3	0
13	393	0	0	0	0	0	0	1	0	1	1	1	2	0	71	97	3	21	0	54	2	8	8	39	1	3	3	16	20	0	8	1	3	0	3	0
14	3493	23	13	12	132	9	4	5	4	45	2	5	0	126	1608	162	255	40	12	29	18	13	132	16	4	29	4	8	5	12	305	15	93	18	50	11
15	3443	269	31	41	50	10	5	1	3	7	32	4	10	0	1642	49	75	59	17	100	4	4	70	5	2	30	0	3	19	33	208	25	189	13	18	1
16	3447	0	0	0	0	230	0	0	0	0	0	0	0	0	182	0	0	0	0	0	0	0	0	0	0	0	0	0	0	0	0	0	0	0	2984	0
17	560	22	2	0	1	0	0	25	0	2	3	0	0	1	252	17	48	26	20	0	4	2	6	6	0	13	0	3	3	0	30	1	4	2	5	0
18	781	0	0	2	18	0	0	2	0	183	1	2	21	2	259	33	6	9	18	6	3	1	8	1	6	4	1	4	0	3	60	6	3	2	4	0
19	1366	1	0	1	0	0	6	0	2	0	3	0	1	0	318	12	1	75	7	7	4	0	8	6	3	1	0	0	11	11	795	0	0	0	2	2
20	2844	41	4	17	27	4	7	3	9	12	5	2	1	4	805	106	69	17	319	81	2	15	44	67	14	20	2	3	18	14	655	29	3	29	16	2
21	3084	0	342	2	337	23	1	2	9	15	6	5	31	11	249	414	29	49	4	7	129	3	8	1	3	2	10	0	15	37	928	74	3	31	26	3
22	959	0	0	0	0	0	1	0	1	0	429	0	42	0	5	0	0	0	0	0	0	0	0	0	0	0	0	0	0	0	455	7	2	0	2	0
23	408	0	0	0	1	0	0	0	0	0	0	1	0	0	12	0	5	1	0	0	4	23	1	1	2	0	9	2	2	0	311	3	2	1	3	1
24	292	0	0	0	0	0	0	0	0	1	0	1	4	0	37	11	2	3	0	0	3	16	0	0	1	0	1	5	4	0	193	6	0	0	1	0
25	462	0	0	0	0	0	0	0	0	0	0	0	0	0	0	0	0	0	0	0	0	0	0	0	0	0	0	0	0	0	445	17	0	0	0	0
26	3003	2	1	3	5	0	5	2	5	3	4	3	8	1	100	38	2	6	5	174	22	94	11	14	0	0	156	3	5	2	2073	75	0	4	23	34

*See full name in supplementary table 2.

Supplementary Table S5. Association between clades and PANGO lineages.

Clade Id	Sequences	PANGO lineage	
		Name	Frequency (%)
1	211	B.1	78.20
2	395	B.1.93	91.65
3	353	B.1	51.27
4	261	B.1	96.17
5	1796	A.1	79.06
6	1685	B	36.26
7	3289	B.2	22.29
8	221	B.1	57.47
9	218	B.1.8	96.33
10	425	B.1	83.53
11	236	B.1	93.22
12	1007	B.1	75.67
13	393	B.1.1	92.88
14	3493	B.1.1	77.64
15	3443	B.1.1	54.28
16	3447	B.1.1.25	96.08
17	560	B.1.1	91.43
18	781	B.1.1	91.68
19	1366	B.1.1	87.48
20	2844	B.1	44.30
21	3084	B.1	42.67
22	959	B.1	64.13
23	408	B.1	90.69
24	292	B.1	88.36
25	462	B.1.21	93.94
26	3003	B.1	53.45

Supplementary Table S6. Sequence distribution in the major clades of GeneBank dataset by collection date groups

Major Clade Id	Sequences	Jan & Before	Feb	Mar	Apr	May	Jun	Jul	Aug
1	75	0	0	0	74	1	0	0	0
2	57	0	0	17	26	8	2	2	2
3	511	1	0	172	181	114	32	7	2
4	86	0	0	13	19	17	15	11	11
5	61	0	0	0	1	1	0	59	0
6	71	0	0	33	38	0	0	0	0
7	246	0	0	67	70	19	35	49	6
8	175	1	0	83	49	8	5	1	0
9	1144	8	11	294	187	129	260	93	45
10	907	2	16	136	179	158	198	63	149
11	463	0	0	182	137	16	14	12	21
12	1465	99	70	823	321	83	56	3	0
13	733	55	125	275	150	24	5	1	0
14	140	0	0	66	42	27	4	1	0
15	81	0	0	50	19	7	2	0	0
16	406	0	0	89	97	70	87	35	28
17	170	1	0	109	48	4	0	2	0
18	68	0	0	28	20	11	4	1	0
19	1342	55	0	0	0	0	99	1188	0
20	1134	37	0	1	0	5	143	948	0
21	582	13	0	0	0	0	42	527	0
22	256	4	0	0	1	36	120	95	0
23	1498	4	7	245	168	282	608	89	9
24	216	0	0	6	36	66	73	14	20

Supplementary Table S7. Sequence distribution in the major clades of GISAID dataset by collection date groups

Major Clade Id	Sequences	Jan & Before	Feb	Mar	Apr	May	Jun	Jul	Aug
1	211	20	1	98	61	16	14	0	0
2	395	0	0	42	280	64	5	0	0
3	353	7	0	81	201	34	14	1	1
4	261	0	2	143	94	14	5	0	0
5	1796	26	62	1060	428	140	76	2	2
6	1685	240	242	923	238	32	6	1	0
7	3289	213	359	1662	841	103	52	21	2
8	221	1	1	178	37	2	0	0	0
9	218	35	0	96	49	21	11	2	0
10	425	21	7	186	130	37	12	24	1
11	236	2	0	49	57	34	44	42	6
12	1007	2	1	497	201	87	68	21	20
13	393	5	0	300	75	3	1	1	0
14	3493	50	3	736	1041	608	370	224	311
15	3443	88	11	607	985	496	224	433	317
16	3447	104	0	1	6	57	346	2690	189
17	560	1	0	148	208	58	47	77	14
18	781	9	1	205	202	52	47	55	146
19	1366	6	1	116	181	293	524	102	116
20	2844	20	6	807	857	356	152	158	301
21	3084	48	24	862	792	532	364	135	282
22	959	1	0	209	180	209	246	111	3
23	408	1	0	212	117	46	19	4	1
24	292	0	3	190	58	37	3	1	0
25	462	0	0	173	149	110	27	3	0
26	3003	21	2	1031	986	537	205	99	53

Supplementary Table S8. Sequence distribution in the major clades of GISAID dataset by host age groups

Major Clade Id	Sequences	Youth	Young Adult	Adult	Senior
1	211	1	15	27	25
2	395	0	0	2	2
3	353	1	8	15	27
4	261	3	15	21	28
5	1796	4	106	138	107
6	1685	24	151	244	338
7	3289	41	436	440	531
8	221	0	3	8	12
9	218	0	25	6	11
10	425	12	49	70	85
11	236	3	13	18	16
12	1007	7	108	136	183
13	393	16	40	82	61
14	3493	50	311	310	317
15	3443	64	370	411	383
16	3447	8	1190	880	687
17	560	7	51	64	74
18	781	10	53	57	37
19	1366	5	39	45	83
20	2844	37	215	342	372
21	3084	38	311	449	492
22	959	25	185	192	314
23	408	3	28	45	59
24	292	0	6	15	16
25	462	0	1	2	2
26	3003	27	264	323	425

Supplementary Table S9. Sequence distribution in the major clades of GISAID dataset by host genders

Major Clade Id	Sequences	Male	Female
1	211	32	38
2	395	3	5
3	353	31	22
4	261	51	31
5	1796	222	158
6	1685	414	371
7	3289	912	636
8	221	12	15
9	218	17	28
10	425	109	114
11	236	36	25
12	1007	269	327
13	393	112	86
14	3493	538	464
15	3443	565	672
16	3447	1326	1272
17	560	109	90
18	781	87	80
19	1366	73	96
20	2844	541	475
21	3084	830	523
22	959	375	369
23	408	84	60
24	292	22	16
25	462	4	3
26	3003	535	527
